# Antenatal care quality and detection of risk among pregnant women: An observational study in Ethiopia, India, Kenya, and South Africa

**DOI:** 10.1371/journal.pmed.1004446

**Published:** 2024-08-27

**Authors:** Catherine Arsenault, Nompumelelo Gloria Mfeka-Nkabinde, Monica Chaudhry, Prashant Jarhyan, Tefera Taddele, Irene Mugenya, Shalom Sabwa, Katherine Wright, Beatrice Amboko, Laura Baensch, Gebeyaw Molla Wondim, Londiwe Mthethwa, Emma Clarke-Deelder, Wen-Chien Yang, Rose J. Kosgei, Priyanka Purohit, Nokuzola Cynthia Mzolo, Anagaw Derseh Mebratie, Subhojit Shaw, Adiam Nega, Boikhutso Tlou, Günther Fink, Mosa Moshabela, Dorairaj Prabhakaran, Sailesh Mohan, Damen Haile Mariam, Jacinta Nzinga, Theodros Getachew, Margaret E. Kruk

**Affiliations:** 1 Department of Global Health, The George Washington University Milken Institute School of Public Health, Washington, DC, United States of America; 2 Department of Family Medicine, Howard College campus, University of KwaZulu-Natal, KwaZulu-Natal, South Africa; 3 Public Health Foundation of India, New Delhi, India; 4 Ethiopian Public Health Institute, Addis Ababa, Ethiopia; 5 Health Economics Research Unit, KEMRI-Wellcome Trust Research Programme, Nairobi, Kenya; 6 Department of Global Health and Population, Harvard T.H. Chan School of Public Health, Boston, Massachusetts, United States of America; 7 Laterite Kenya, Nairobi, Kenya; 8 School of Nursing and Public Health, University of KwaZulu-Natal, Durban, South Africa; 9 Swiss Tropical and Public Health Institute, University of Basel, Basel, Switzerland; 10 Department of Obstetrics and Gynecology, University of Nairobi, Nairobi, Kenya; 11 Department of Health Systems Management and Health Policy, School of Public Health, College of Health Sciences, Addis Ababa University, Addis Ababa, Ethiopia; 12 Department of Public Health Medicine, Howard College campus, University of KwaZulu-Natal, KwaZulu-Natal, South Africa; 13 Department of International Public Health, Liverpool School of Tropical Medicine, Liverpool, United Kingdom

## Abstract

**Background:**

Antenatal care (ANC) is an essential platform to improve maternal and newborn health (MNH). While several articles have described the content of ANC in low- and middle-income countries (LMICs), few have investigated the quality of detection and management of pregnancy risk factors during ANC. It remains unclear whether women with pregnancy risk factors receive targeted management and additional ANC.

**Methods and findings:**

This observational study uses baseline data from the MNH eCohort study conducted in 8 sites in Ethiopia, India, Kenya, and South Africa from April 2023 to January 2024. A total of 4,068 pregnant women seeking ANC for the first time in their pregnancy were surveyed. We built country-specific ANC completeness indices that measured provision of 16 to 22 recommended clinical actions in 5 domains: physical examinations, diagnostic tests, history taking and screening, counselling, and treatment and prevention. We investigated whether women with pregnancy risks tended to receive higher quality care and we assessed the quality of detection and management of 7 concurrent illnesses and pregnancy risk factors (anemia, undernutrition, obesity, chronic illnesses, depression, prior obstetric complications, and danger signs). ANC completeness ranged from 43% in Ethiopia, 66% in Kenya, 73% in India, and 76% in South Africa, with large gaps in history taking, screening, and counselling. Most women in Ethiopia, Kenya, and South Africa initiated ANC in second or third trimesters. We used country-specific multivariable mixed-effects linear regression models to investigate factors associated with ANC completeness. Models included individual demographics, health status, presence of risk factors, health facility characteristics, and fixed effects for the study site. We found that some facility characteristics (staffing, patient volume, structural readiness) were associated with variation in ANC completeness. In contrast, pregnancy risk factors were only associated with a 1.7 percentage points increase in ANC completeness (95% confidence interval 0.3, 3.0, *p*-value 0.014) in Kenya only. Poor self-reported health was associated with higher ANC completeness in India and South Africa and with lower ANC completeness in Ethiopia. Some concurrent illnesses and risk factors were overlooked during the ANC visit. Between 0% and 6% of undernourished women were prescribed food supplementation and only 1% to 3% of women with depression were referred to a mental health provider or prescribed antidepressants. Only 36% to 73% of women who had previously experienced an obstetric complication (a miscarriage, preterm birth, stillbirth, or newborn death) discussed their obstetric history with the provider during the first ANC visit. Although we aimed to validate self-reported information on health status and content of care with data from health cards, our findings may be affected by recall or other information biases.

**Conclusions:**

In this study, we observed gaps in adherence to ANC standards, particularly for women in need of specialized management. Strategies to maximize the potential health benefits of ANC should target women at risk of poor pregnancy outcomes and improve early initiation of ANC in the first trimester.

## Abstract 2

Catherine Arsenault and co-workers leverage international survey data collected at first antenatal care visits in Ethiopia, Kenya, India and South Africa to help understand where and why gaps may exist.

## Author summary

**Why was this study done?:**

- Past research has revealed gaps in the content and quality of antenatal care (ANC) in some settings.
- However, few studies have explored whether women at risk receive additional care during pregnancy.
- In general, evidence on the quality of risk detection during pregnancy in low-resource settings is limited.

**What did the researchers do and find?:**

- We used the baseline survey of the eCohort, a study conducted in Ethiopia, Kenya, India, and South Africa, that enrolled 4,068 women during their first ANC visit and involved physical health assessments and patient interviews.
- We found large gaps in the provision of essential clinical actions during the first ANC visit where only 43% of essential clinical actions were provided in Ethiopia compared to 66% in Kenya, 73% in India, and 76% in South Africa.
- We found a high prevalence of anemia, chronic illnesses, undernutrition, obesity, and prior obstetric complications which are risk factors for poor pregnancy outcomes.
- We found little to no association between the presence of risk factors and the completeness of the first ANC visit.

**What do these findings mean?:**

- Efforts to improve the detection and management of pregnancy risks as well as improvements in adherence to ANC standards are required.
- Policymakers should interrogate the structural factors, such as preservice training, supervision, facility infrastructure, or continuing education, responsible for poor performance.
- Some limitations may affect our results. For example, most of the data on care completeness was based on patient self-reports which may be influenced by the participants’ education or by social desirability bias.

## Introduction

Efforts remain to improve maternal and newborn health (MNH) in low- and middle-income countries (LMICs). Globally, 88% of pregnant women now access skilled antenatal care (ANC) at least once during their pregnancy [1]. However, despite near universal coverage with ANC, gaps in adherence to clinical standards mean that use of this platform does not always yield the desired outcomes [2–7]. According to the World Health Organization (WHO), ANC should encompass 4 key components: risk identification; prevention and management of pregnancy-related or concurrent diseases; and health education and health promotion [5]. This platform plays a crucial role in addressing prevalent health risks in LMICs, including anemia, malnutrition, infectious diseases, chronic illnesses, or prenatal depression [5].

Anemia during pregnancy can have important consequences for mothers and newborns. Anemia increases the risk of postpartum hemorrhage, it can lead to intrauterine growth restriction, low birth weight, preterm birth, and neonatal death, in addition to affecting the overall well-being of the pregnant woman and increasing her susceptibility to infections [8,9]. Similarly, being underweight during pregnancy increases the risk of birth complications and can lead to fetal growth restrictions, preterm birth, and developmental delays [10,11].

ANC provides an opportunity to improve maternal nutrition through counseling, protein-rich food supplementation, and micronutrient supplementation with iron and folic acid (IFA), calcium, and vitamin A. Iron is essential to prevent anemia while calcium supplementation in populations with low dietary calcium intake has been shown to reduce the risk of preeclampsia and preterm birth [12]. Depression or infectious diseases during pregnancy (e.g., syphilis, HIV, malaria, hepatitis B and C) also pose several risks to the woman and baby and should be addressed during ANC [13–17]. Finally, fetal and pregnancy factors that may require specialized obstetric care, such as twin pregnancies, breech presentation, placenta previa, abruptio placentae, and hypertension disorders are often detectable in pregnancy but not infrequently missed putting mother and baby at risk of mortality [17–21].

As many modifiable risks can be detected and addressed in pregnancy, the detection and timely referral of such pregnancies is a central task of ANC. Antenatal risk assessment requires detailed history taking (obstetric, medical, and surgical history), systematic physical examinations, diagnostic tests, and imaging. Women with pregnancy risk factors may require additional assessments and treatments, and/or specialized delivery arrangements. Several studies have assessed the content of ANC consultations in sub-Saharan Africa and India and have shown gaps in the provision of essential clinical actions such as blood pressure monitoring and urine or blood testing [2–4,18,22,23]. However, there is little evidence on the performance of health systems in detecting and addressing specific health problems during pregnancy [6,7]. It also remains unclear whether women at greater risk will receive additional care during pregnancy compared to other women.

In this paper, we describe the completeness of first ANC visits for pregnant women in 4 countries and we investigate whether women with pregnancy risk factors tend to receive higher quality care. We also describe the quality of pregnancy risk detection and management during the first ANC visit.

## Methods

### Data source

This analysis uses data from the MNH eCohort, a longitudinal health system quality study that enrolled women face-to-face during their first ANC visit and followed them over the phone through pregnancy, delivery, and the postnatal period. The study was implemented in 4 countries: Ethiopia, India, Kenya, and South Africa. In the 3 African countries, 2 sites were selected for the study: 1 predominantly rural and 1 urban. In India, the study was conducted in 2 districts from 2 distinct states (Sonipat district in Haryana and Jodhpur in Rajasthan) where both urban and rural health facilities were selected. The study sites are described in **Table A in S1 Information**. In each country, between 21 and 29 health facilities were selected for recruitment of pregnant women. To guide our sampling strategy in each site, we used Demographic and Health Surveys and health management information system data to estimate the proportion of first ANC visits that take place across 4 types of facilities: public versus private facilities and primary care facilities versus hospitals. We aimed to enroll 500 women in each site (1,000 per country) and no less than 50 women per relevant facility strata. While the Ethiopia and Kenya samples included all 4 facility types, the India sample included only public facilities (both primary and secondary) and the South Africa sample included only public primary care facilities. With the help of health providers, data collectors identified pregnant women who were presenting to the study health facilities to receive their first ANC visit. All women aged 15 and above (or 18 in India) seeking care for the first time in their pregnancy were eligible for inclusion in the study if they were planning to continue to reside in the study site over the course of the study. This analysis uses data from the baseline survey conducted face-to-face at health facilities in the 4 countries from April 2023 to January 2024. The baseline survey also included health assessments (blood pressure, weight, height, mid-upper arm circumference (MUAC) measurement, and a rapid test for anemia), a brief health facility assessment, and an interview with the facility manager. More details on the methodology and survey development process for the eCohort are available from the corresponding author.

### Completeness of the first ANC visit

The baseline survey was conducted immediately after the first ANC visit. Women were asked to report on the content of the visit. Using global and national ANC guidelines, and maternal health records or patient-held handbooks, we built country-specific ANC completeness indices that measured provision of recommended clinical actions in 5 domains: physical examinations, diagnostic tests, history taking and screening, counselling, and treatment and prevention [5,24–31]. The indices varied slightly across the 4 countries due to differences in national recommendations. They included 16 items in India, 19 items in South Africa, 20 items in Kenya, and 22 items in Ethiopia which are described in **Box 1 and Table B in S1 Information**. We computed completeness indices for each woman as the average of the total required items she received during the visit, where each item was weighed equally. For example, although provision of calcium supplements is required in Ethiopia, India, and South Africa to reduce the risk of preeclampsia, it is not required in Kenya [26]. The indices also varied according to the woman’s gestational age during her first visit as certain clinical actions only apply to women in later stages of pregnancy.

### Antenatal risk factors

Our primary independent variable of interest was the presence of pregnancy risk factors. We derived conditions used to risk-stratify a pregnancy from ANC guidelines and the literature [21,24–31]. Conditions for classification as a “high-risk” pregnancy included anemia, hypertension, diabetes, a history of any systemic illness(es), a body mass index (BMI) ≥30, mother’s age <20 or >35, and obstetric risk factors such as multiple pregnancies, previous caesarean sections, or a history of (other) obstetric complications including stillbirths, premature births, neonatal deaths, or postpartum hemorrhages. For our analysis, we also included being underweight (MUAC <23 cm or BMI <18.5) as a potential risk factor given extensive evidence on its association with poor pregnancy outcomes [11,32,33]. The hemoglobin level to assess anemia were extracted from the maternal health card. If it was missing from the card or if the card was not available, data collectors performed a rapid hemoglobin test. Hypertension was either self-reported (if previously diagnosed) or identified by the data collector by measuring the blood pressure during the baseline interview and defined as systolic blood pressure ≥140 mmHg or diastolic pressure ≥90 mmHg.

### Statistical analysis

We explored associations between woman-, facility-, and site-level characteristics and the completeness of the first ANC visit. In addition to the presence of potential risk factors described above, women characteristics included demographics (education, wealth, health literacy, marital status), self-reported health, experience of danger signs, primiparity and whether the pregnancy had been intended. Health literacy was based on 6 health knowledge questions adapted from the Indian Health and Human Development Survey (**Table C in S1 Information)** [34]. Self-reported health was based on the woman’s rating of her own health measured on a Likert scale (excellent, very good, good, fair poor). The potential danger signs included having experienced any of the following at least once in the pregnancy: severe or persistent headaches, vaginal bleeding of any amount, fever, severe abdominal pain (not just discomfort), convulsions or seizures, repeated fainting, or loss of consciousness.

Facility characteristics included facility type, ownership, staffing, volume of ANC visits, and service readiness. ANC volumes (average number of ANC visits conducted each month over the prior year) were extracted from facility registers. The service readiness score was based on WHO service availability and readiness assessment (SARA) guidelines [35]. The service readiness score included presence of basic amenities (e.g., facility has water, sanitation, electricity), basic equipment required for MNH care (e.g., blood pressure cuff, adult scale, and ultrasound), and diagnostic capacity (e.g., syphilis and HIV rapid diagnostic tests). These are described in **Tables D and E in S1 Information**. We used country-specific multilevel linear regression models to assess associations between woman- and facility-level characteristics and the completeness of the first ANC visit. All models included a random intercept for the facility, a fixed effect for the site, and robust standard errors. Potential multicollinearity between the independent variables was assessed using variance inflation factors. The regression analyses were repeated using a categorical variable representing the number of risk factors each woman had: no risk factor, 1 risk factor, 2 risk factors, or 3 or more risk factors.

Finally, to further explore the quality of pregnancy risk detection and management during ANC, we conducted a sub-analysis focused on women with one of the following conditions: anemia, chronic illnesses, prior obstetric complications, danger signs, undernutrition, obesity, or depression. Depression was assessed using the Patient Health Questionnaire– 9 (PHQ9) using a cutoff of 5 for mild to severe depression [36]. Women with any of the above condition were asked to report whether the condition had been discussed at any point during the first ANC visit, or whether they had been screened for the condition, and whether the condition had been managed through relevant treatments, counseling, or referral.

Given important differences in macroeconomic contexts, health system capacities and epidemiologic profiles, all analyses were done separately in each of the 4 countries and country-specific estimates were compared. A complete case analysis was performed, excluding any observations with missing values on variables of interest.

### Ethical approval

The study protocol was reviewed and approved by the Institutional Review Boards (IRB) of the Harvard T.H. Chan School of Public Health (protocol #IRB22-0487), the Kenya Medical Research Institute (protocol number KEMRI/SERU/CGMR-C/4226), the Ethiopian Public Health Institute (protocol number EPHI-IRB-448-2022), the University of KwaZulu-Natal (protocol number BREC/00004645/2022), and the Public Health Foundation of India (protocol number TRC-IEC 495/22). Formal informed consent was obtained from all adult study participants or emancipated minors or from formal guardians or next-of-kins for study participant under the age of 18 years old in accordance with local regulations.

## Results

Our analysis included a total of 4,068 pregnant women seeking care for the first time in pregnancy (**Table 1**). Mean age ranged from 24 to 27 years old across the 8 sites. The proportion of pregnant adolescents enrolled in the study (<20 years old) ranged from 5% in the Ethiopian urban site to 22% in the rural South African site. There were notable differences in health literacy. Only 12% of Indian women correctly answered the 6 health knowledge questions compared to 27% in Ethiopia, 33% in South Africa, and 45% in Kenya. Only 17% of South African women reported that their pregnancy had been intended while between 62% of women in Kenya and 91% of women in India had intended to become pregnant. South Africa also had the lowest proportion of married women (or living with a partner). In India and South Africa, a higher proportion of women were in the first trimester of pregnancy (51% and 41%, respectively). Conversely, in Ethiopia and Kenya, the majority of women initiated ANC in their second trimester (63% in both countries). One in 5 women in Kenya initiated ANC in the third trimester of pregnancy.

**Table 1:** Characteristics of pregnant women included in the analysis.

	Ethiopia		Kenya		India		South Africa	
	Rural	Urban	Rural	Urban	Rural	Urban	Rural	Urban
	*N* = 508	*N* = 492	*N* = 504	*N* = 498	*N* = 365	*N* = 657	*N* = 516	*N* = 528
Demographics	N (%)	N (%)	N (%)	N (%)	N (%)	N (%)	N (%)	N (%)
Age mean (SD)	24.4 (4.7)	26.4 (4.8)	26.2 (6.6)	27.5 (5.9)	24.1 (3.7)	24.7 (4.2)	26.1 (6.6)	27.4 (6.3)
Adolescents (aged under 20)	60 (11.8%)	26 (5.3%)	89 (17.7%)	31 (6.2%)	32 (8.8%)	46 (7.0%)	115 (22.3%)	58 (11.0%)
Completed secondary school	82 (16.1%)	139 (28.3%)	221 (43.8%)	364 (73.1%)	194 (53.2%)	339 (51.7%)	328 (63.6%)	409 (77.6%)
Answers health literacy questions correctly ^a^	119 (23.4%)	147 (29.9%)	237 (47.0%)	211 (42.4%)	57 (15.6%)	70 (10.7%)	84 (16.3%)	263 (49.8%)
Wealth tertiles ^b^								
Poorest	274 (54.9%)	54 (11.2%)	296 (59.1%)	37 (7.5%)	117 (32.1%)	226 (34.7%)	137 (26.6%)	127 (24.1%)
Middle	171 (34.3%)	155 (32.2%)	162 (32.3%)	259 (52.3%)	151 (41.4%)	257 (39.5%)	195 (37.8%)	216 (41.0%)
Richest	54 (10.8%)	273 (56.6%)	43 (8.6%)	199 (40.2%)	97 (26.6%)	168 (25.8%)	184 (35.7%)	184 (34.9%)
Married or partnered	496 (97.6%)	476 (96.9%)	368 (73.0%)	413 (83.6%)	365 (100.0%)	656 (99.8%)	50 (9.7%)	76 (14.4%)
**Health status**								
Rates own health as poor or fair ^c^	75 (14.8%)	95 (19.3%)	29 (5.8%)	57 (11.4%)	25 (6.8%)	73 (11.1%)	58 (11.2%)	24 (4.6%)
Depression (PHQ ≥5)	125 (24.6%)	122 (24.8%)	106 (21.0%)	90 (18.1%)	33 (9.0%)	59 (9.0%)	241 (46.7%)	129 (24.4%)
Reports at least 1 danger sign ^d^	150 (29.5%)	92 (18.7%)	100 (19.8%)	131 (26.3%)	40 (11.0%)	91 (13.9%)	266 (51.7%)	218 (41.4%)
**Characteristics of current pregnancy**								
Primipara	149 (29.3%)	178 (36.2%)	157 (31.2%)	177 (35.5%)	180 (49.3%)	321 (48.9%)	182 (35.3%)	175 (33.1%)
Pregnancy was intended	348 (68.5%)	380 (77.4%)	294 (58.8%)	316 (64.5%)	336 (92.1%)	592 (90.5%)	87 (16.9%)	94 (17.9%)
Estimated gestational age in trimester at first visit:							
First trimester 0–12 weeks	137 (27.7%)	167 (34.4%)	60 (11.9%)	94 (18.9%)	186 (51.0%)	337 (51.3%)	181 (35.1%)	246 (46.7%)
Second trimester 13–26 weeks	328 (66.3%)	287 (59.2%)	311 (61.7%)	323 (64.9%)	162 (44.4%)	283 (43.1%)	269 (52.2%)	238 (45.2%)
Third trimester 27–42 weeks	30 (6.1%)	31 (6.4%)	133 (26.4%)	81 (16.3%)	17 (4.7%)	37 (5.6%)	65 (12.6%)	43 (8.2%)

In Ethiopia, the rural site is East Shewa Zone, and the urban site is Adama Town, both in Oromia region. In Kenya, the rural site is Kitui County, and the urban site is Kiambu county. In India, rural and urban facilities were selected from Sonipat and Jodhpur districts in the states of Haryana and Rajasthan, respectively. In South Africa, the rural site is Nongoma local municipality, and the urban site is the city of uMhlathuze, both in the Province of KwaZulu-Natal.

^a^Proportion of women who responded correctly to 6 questions aimed at testing their health knowledge (described in **Table C in S1 Information**).

^b^Based on a wealth index constructed using principal component analysis and based on questions related to asset ownership and construction materials of the house.

^c^Compared to excellent, very good, or good.

^d^Experienced at least one of the following in the pregnancy so far: severe or persistent headache, vaginal bleeding of any amount, a fever, severe abdominal pain (not just discomfort), convulsions or seizures, repeated fainting, or loss of consciousness.

**Fig 1:**
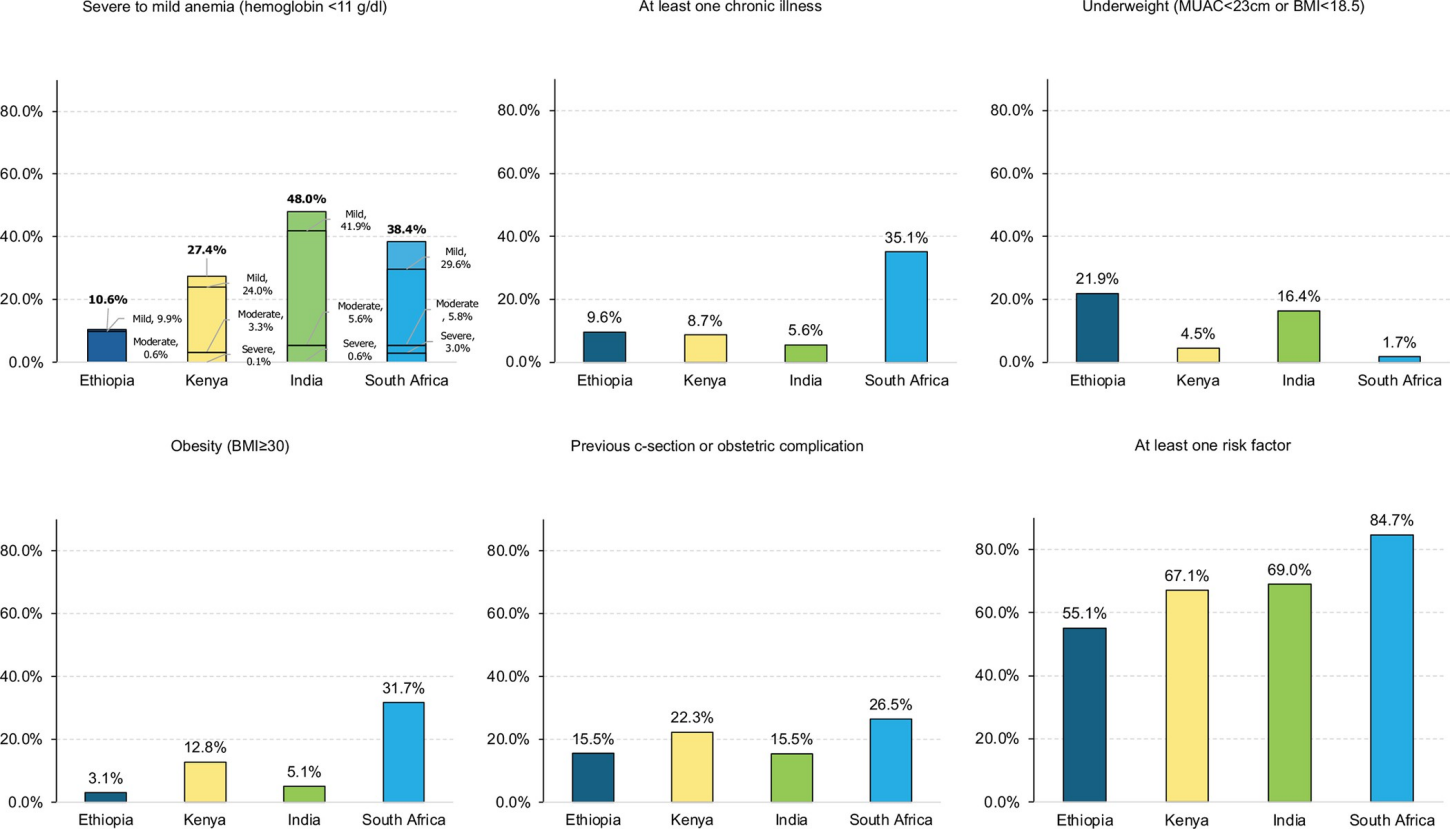
Prevalence of pregnancy risk factors during the first ANC visit in 4 countries. (1) Hemoglobin (Hb) levels were extracted from the maternal health card at enrollment or, if not available in the card, a rapid Hb test was conducted by the eCohort data collector. Severe anemia defined as Hb <7g/dL, moderate anemia is 7–8.9 g/dL, and mild anemia is 9–10.9 g/dL. (2) Chronic illnesses included high blood pressure identified during enrollment in the study (systolic blood pressure ≥140 mmHg or diastolic pressure ≥90 mmHg) and self-reported chronic illnesses (diabetes, hypertension, cardiac disease, HIV, mental health disorder, schizophrenia, renal disorder, asthma, chronic pelvic inflammatory disease, epilepsy, seizures, ovarian cysts, peptic ulcer disease, cancer, thyroid disease, uterine myoma, genital tract abnormalities, hemoglobinopathies, tuberculosis, kidney failure, fibroids, a history of stroke). (3, 4) Nutritional assessments (weight, height, and MUAC measurement) were performed by eCohort data collectors at enrollment. Undernutrition may be underestimated, and obesity overestimated as the BMI was assessed during pregnancy. (5) Proportion of respondents who report at least 1 previous cesarean section, or complication (stillbirth, neonatal death, preterm birth (baby born more than 3 weeks before the due date), or postpartum hemorrhage). (6) Proportion of women who have at least 1 risk factor: anemia (Hb <11 g/dL), chronic illnesses, underweight, obesity, prior obstetric complication, known multiple pregnancy, age >35 or <20. ANC, antenatal care; BMI, body mass index; MUAC, mid-upper-arm circumference.

**Fig 1** shows the proportion of women with potential pregnancy risk factors. The prevalence of anemia (hemoglobin level <11 g/dL) was highest in India, while chronic illnesses were most common in South Africa where HIV was the most commonly reported chronic illness. Approximately one in 5 women in Ethiopia and India were underweight while 32% (329/1038) of women in South Africa had a BMI ≥30. Between 16% of women in Ethiopia (155/1,000) and India (158/1,022) and 27% of women in South Africa (277/1,044) had a prior obstetric complication or c-section.

**Fig 2:**
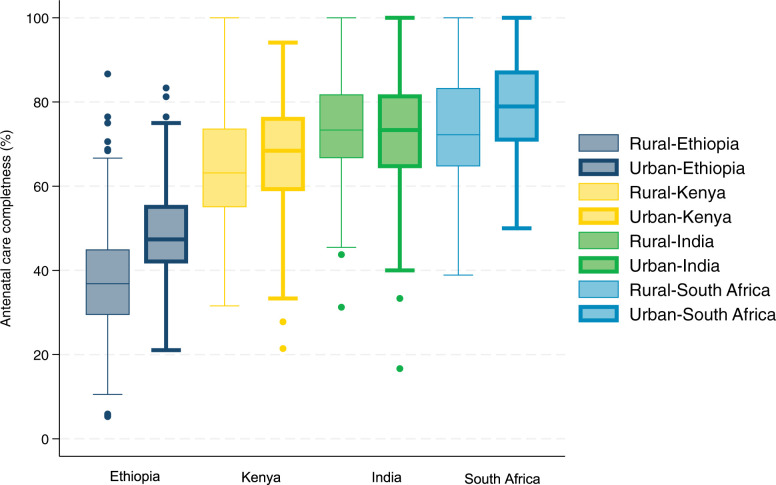
Completeness of the first ANC visit. In Ethiopia, the rural site is East Shewa Zone, and the urban site is Adama Town, both in Oromia region. In Kenya, the rural site is Kitui County, and the urban site is Kiambu county. In India, rural and urban facilities were selected from Sonipat and Jodhpur districts in the states of Haryana and Rajasthan, respectively. In South Africa, the rural site is Nongoma local municipality, and the urban site is the city of uMhlathuze, both in the Province of KwaZulu-Natal. The ANC completeness score measures provision of 16 to 22 recommended clinical actions in 5 domains: physical examinations, diagnostic tests, history taking and screening, counselling, and treatment and prevention. Details of the score is presented in Box 1 and Table F in S1 Information. ANC, antenatal care.

**Fig 3:**
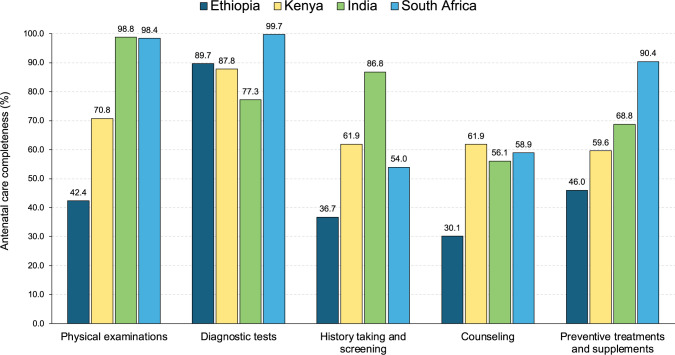
Completeness of first ANC visits by care category. Physical examinations include measurement of blood pressure, weight, height (Ethiopia, Kenya, South Africa), and MUAC (Ethiopia, Kenya, South Africa). Diagnostic testing includes blood tests (blood draw or finger prick), urine tests, and ultrasounds (for women in the third trimester, Ethiopia, India, Kenya). History taking and screening includes assessing the date of the last menstrual period, screening for depression (Ethiopia, Kenya, South Africa), screening for danger signs (Ethiopia, South Africa), and discussing previous pregnancies (among multiparous women). Counseling covered nutrition, exercise (Ethiopia, Kenya, South Africa), signs of pregnancy complications to look out for, birth preparedness, counseling on the need to return for follow-up ANC visits and providing an estimated due date for women in second or third trimester. Treatments and preventive care included IFA supplements, calcium supplements for women in second or third trimester (Ethiopia, India, and South Africa), deworming medication for women in second or third trimester (Ethiopia, India, Kenya), tetanus toxoid vaccination (among women not already protected against tetanus), and provision of insecticide treated bed nets in malaria endemic sites among women who did not already have a bed net (Ethiopia and Kenya). ANC, antenatal care; IFA, iron and folic acid; MUAC, mid-upper arm circumference.

The completeness of first ANC visits according to national standards is shown in **Fig 2 and Table F in S1 Information**. Women in Ethiopia received only 43% of recommended clinical actions on average, compared to 66% in Kenya, 73% in India, and 76% in South Africa. Performance was highest for diagnostic testing (blood test, urine test, and ultrasound) and physical examinations (blood pressure, weight, height, and MUAC) and lowest for counselling and history taking and screening (**Fig 3**). The proportion of preventive treatments received was low (46% to 69%) in 3 countries and but was 90% in South Africa. The clinical actions least commonly performed included provision of deworming medication for women in second or third trimesters (7% on average across 3 countries where they are required), screening for depression (15% on average across 3 countries), and screening for danger signs (27% on average in Ethiopia and South Africa). Only one in 3 women in their third trimester seeking care for the first time in their pregnancy received an ultrasound. Among the preventive treatments assessed, IFA supplementation and tetanus vaccination were relatively high (90% and 75%, respectively, on average across the 4 countries).

Results from the country-specific multilevel regression analyses are shown in **Table 2**. Associations between the presence of risk factors and ANC completeness were minimal and only statistically significant in Kenya where having at least 1 risk factor was associated with a 1.7 percentage-point (%-point) increase in ANC completeness (95% confidence interval (CI) 0.3 to 3.0). Results from the analysis using the number of risk factors as the independent variable were similar (**Tables G and H in S1 Information**).

**Table 2:** Results of linear multilevel regression models for the completeness of the first ANC visit in four countries.

		Ethiopia				Kenya				India				South Africa			
		Coeff	LCL	UCL	*p*-value	Coeff	LCL	UCL	*p*-value	Coeff	LCL	UCL	*p*-value	Coeff	LCL	UCL	*p*-value
**Risk factors**																	
Has at least 1 pregnancy risk^**a**^		0.82	−0.59	2.22	0.254	1.65	0.33	2.97	0.014	0.55	−0.90	2.00	0.459	0.30	−1.94	2.53	0.794
**Demographics**																	
Age																	
	20–34	*ref*.				*ref*.				*ref*.				*ref*.			
	<20	0.18	−2.53	2.88	0.898	−2.06	−4.54	0.42	0.103	−0.78	−2.49	0.92	0.369	0.59	−1.70	2.88	0.613
	35+	−2.06	−5.14	1.01	0.189	0.41	−0.79	1.60	0.505	−2.20	−6.46	2.06	0.311	0.80	−0.82	2.43	0.332
Completed secondary school		0.57	−1.35	2.48	0.561	1.57	0.27	2.87	0.018	−1.36	−2.47	−0.26	0.016	0.88	−0.58	2.35	0.239
Answers health literacy questions correctly		0.58	−1.27	2.43	0.539	0.28	−1.18	1.74	0.708	0.74	−1.08	2.55	0.426	1.32	−0.02	2.66	0.053
Wealth																	
	Poorest	*ref*.				*ref*.				*ref*.				*ref*.			
	Middle	1.46	−0.94	3.87	0.233	0.74	−1.00	2.48	0.406	0.30	−1.02	1.61	0.658	1.62	−0.20	3.43	0.081
	Richest	2.86	0.31	5.42	0.028	0.12	−2.92	3.15	0.940	1.16	−0.36	2.68	0.134	0.97	−0.76	2.70	0.274
Reports at least 1 danger sign^**b**^		1.57	0.14	2.99	0.032	−1.15	−2.72	0.42	0.150	0.55	−1.28	2.38	0.555	0.97	−0.39	2.33	0.162
Rates own health as poor or fair		−1.65	−2.95	−0.35	0.013	−0.48	−3.25	2.30	0.737	2.49	0.86	4.11	0.003	2.48	0.14	4.82	0.038
Primiparous		0.75	−0.71	2.20	0.316	−2.91	−4.56	−1.25	0.001	−1.38	−2.87	0.10	0.068	−1.02	−2.58	0.54	0.198
Pregnancy was intended		0.47	−1.49	2.42	0.640	1.49	0.63	2.34	0.001	0.76	−1.17	2.68	0.440	0.56	−1.38	2.50	0.570
**Facility characteristics** ^**c**^																	
Private		0.92	−3.19	5.03	0.660	2.86	−6.18	11.89	0.535								
Secondary facility		−4.79	−9.67	0.09	0.055	4.96	−2.19	12.11	0.174	2.26	−2.92	7.44	0.392				
Service readiness score^**d**^																	
	Low	*ref*.				*ref*.				*ref*.				*ref*.			
	Middle	−1.39	−7.42	4.63	0.651	7.87	−5.98	21.72	0.266	−1.94	−7.03	3.16	0.457	4.56	0.89	8.24	0.015
	High	−1.17	−6.97	4.63	0.694	3.56	−13.97	21.09	0.691	−4.22	−9.77	1.33	0.136	0.85	−4.02	5.73	0.731
Number of staff providing obstetric care^**e**^																	
	Low	*ref*.				*ref*.				*ref*.				*ref*.			
	Middle	−5.56	−12.77	1.64	0.130	−1.32	−10.76	8.11	0.783	−4.73	−9.40	−0.07	0.047	−3.29	−7.53	0.95	0.128
	High	11.84	4.52	19.17	0.002	−5.65	−13.92	2.61	0.180	−3.37	−7.82	1.08	0.138	1.79	−7.83	11.41	0.716
Number of ANC visits per month^**f**^																	
	Low									*ref*.				*ref*.			
	Middle									−0.26	−4.61	4.09	0.906	−5.29	−9.40	−1.18	0.012
	High									−0.70	−5.89	4.49	0.791	−5.27	−14.59	4.05	0.268
**Site**																	
Rural		*ref*.				*ref*.				*ref*.				*ref*.			
Urban		−2.53	−9.47	4.41	0.475	0.02	−7.91	7.95	0.995	−1.26	−4.76	2.24	0.482	2.05	−2.14	6.25	0.338
**N**		**980**				**984**				**1012**				**1035**			
Intercept		39.39	34.14	44.63	<0.001	60.21	53.73	66.68	<0.001	78.39	72.37	84.41	<0.001	74.94	68.65	81.22	<0.001

Coeff are the linear regression model coefficients for each independent variable. LCL are lower confidence limits of the 95% CI, UCL are upper confidence limits of the 95% CI.

^**a**^Has at least 1 risk factor: anemia (Hb <11 g/dL), chronic illnesses, underweight, obesity, prior obstetric complication, known multiple pregnancy, age >35 or <20.

^**b**^Experienced at least one of the following in the pregnancy so far: severe or persistent headache, vaginal bleeding of any amount, a fever, severe abdominal pain (not just discomfort), convulsions or seizures, repeated fainting, or loss of consciousness.

^**c**^Facility types, service readiness, staffing and volume of ANC visits are described in **Tables D and E in S1 Information**.

^**d**^Low service readiness score was 0.28 to 0.51 in Ethiopia, 0.58 to 0.74 in Kenya, 0.17 to 0.47 in India, and 0.67 to 0.74 in South Africa. Middle category of service readiness was 0.53 to 0.69 in Ethiopia, 0.77 to 0.85 in Kenya, 0.60 to 0.72 in India, and 0.77 to 0.83 in South Africa. High service readiness score was 0.72 to 0.97 in Ethiopia, 0.88 to 0.97 in Kenya, 0.75 to 0.88 in India, and 0.83 to 0.88 in South Africa.

^**e**^Low number of staff providing obstetric care was 1 to 2 in Ethiopia, 4 to 9 in Kenya, 5 to 12 in India, and 1 to 4 in South Africa. Middle category of staff providing obstetric care was 3 in Ethiopia, 11 to 24 in Kenya, 14 to 18 in India, and 5 to 19 in South Africa. High number of staff providing obstetric care was 4 to 14 in Ethiopia, 28 to 66 in Kenya, 20 to 133 in India and 20 to 28 in South Africa.

^**f**^Low number of ANC visits per month was in 7–24 in India and 20–149 in South Africa. Middle category of ANC visits per month was 30–63 in India and 168–465 in South Africa. High number of ANC visits per month was 65–903 in India and 480–776 in South Africa.

Other individual-level characteristics also had small associations with ANC completeness. In Kenya, ANC completeness was lower for primiparous women (−2.9, 95% CI −4.6, −1.3) and higher for women who had intended to become pregnant (1.5, 95% CI 0.6 to 2.3). Wealthier women tended to receive more complete care, but this was only statistically significant in Ethiopia where ANC completeness was 2.9%-point higher in the richest tertile (95% CI 0.3, 5.4). In Ethiopia, reporting a danger sign was associated with a 1.6%-point increase in ANC completeness (95% CI 0.1 to 3.0). Poor self-reported health was associated with higher ANC completeness in India and South Africa and lower ANC completeness in Ethiopia.

In contrast, some facility characteristics had larger associations with ANC completeness. In Ethiopia, ANC completeness was 11.8%-point higher in health facilities that had at least 4 health care staff providing ANC (95% CI 4.5, 19.2). In South Africa, facilities with a higher service readiness score had better ANC completeness and those with the highest volume of ANC visits per month had a 5.3%-point lower ANC completeness (95% CI −9.4, −1.2). Surprisingly in India, better staffed health facilities had lower ANC completeness. We found no statistically significant differences between public and private facilities in Ethiopia and Kenya.

**Fig 4:**
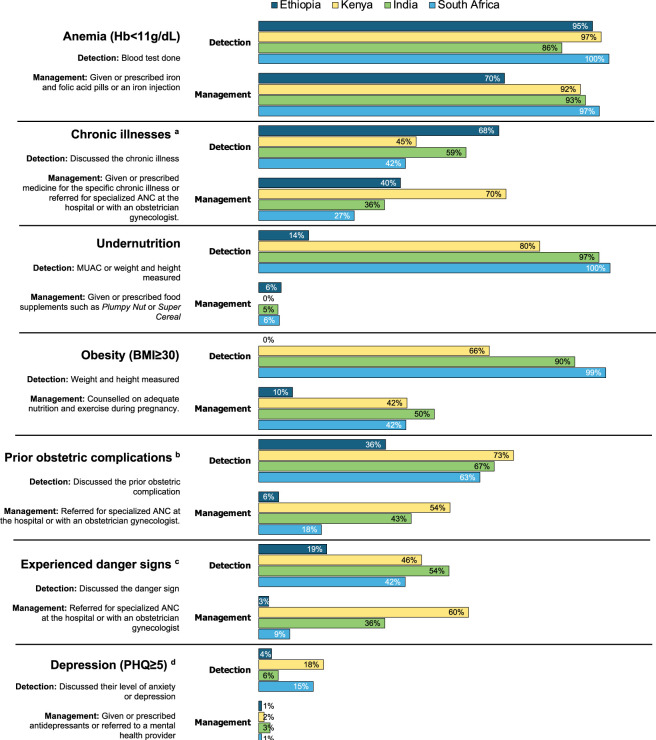
Proportion of women with seven potential risk factors who received basic care. (a) For women with a chronic illness: diabetes, hypertension, a cardiac problem, a mental health problem, or HIV. The prevalence of these 5 conditions varies widely across the 4 countries. (b) For women with a prior obstetric complication: a prior late miscarriage, stillbirth, preterm birth, or neonatal death. (c) Women who report experiencing at least one of the following in the pregnancy so far: severe or persistent headaches, vaginal bleeding of any amount, a fever, severe abdominal pain (not just discomfort), convulsions or seizures, repeated fainting, or loss of consciousness. (d) Depression assessed using the PHQ9. PHQ9, Patient Health Questionnaire– 9. BMI, body mass index; Hb, hemoglobin.

**Fig 4** shows the detection and management of 7 common pregnancy risk factors. Performance was poorest for prenatal depression. Although 22% (905/4,068) of women in our study had depression according to the PHQ9, only between 4% (9/247) and 18% (36/195) of women with depression (in Ethiopia and Kenya, respectively) were screened for depression during the first ANC visit and less than 3% were referred to a mental health provider or prescribed antidepressants. Despite having the highest prevalence of undernutrition (22%, 218/997), Ethiopia had the lowest levels of detection of undernutrition where only 14% (31/218) of women had their MUAC measured. A maximum of 6% (14/217) of underweight pregnant women received or were prescribed food supplements across the 4 countries. Management of obesity was also poor in all 4 countries where only 10% (3/31) to 50% (25/50) of women with a BMI ≥30 (in Ethiopia and India, respectively) received counseling on nutrition and exercise. Referral to hospital (or with a specialist) for women experiencing danger signs or those with prior obstetric complications was lowest in Ethiopia (<6%, 6/105) and ranged from 9% in South Africa (43/484) to 60% (138/231) in Kenya.

## Discussion

In this paper, we described the completeness of 4,068 first ANC visits in 4 countries and explored associations between the presence of pregnancy risk factors and the completeness of ANC. We also described the quality of detection and management of concurrent illnesses and risk factors for poor health outcomes. We found gaps in the provision of essential clinical actions during ANC where only 43% to 76% of essential care was received on average. In Ethiopia, India, and South Africa, we found no evidence that women with pregnancy risks would receive more complete care. In Kenya, presence of risk factors was associated with only a 1.7 percentage points increase in ANC completeness. In addition, we found poor levels of screening for malnutrition, depression, chronic illnesses, or dangerous symptoms. Prior obstetric complications, which are important predictors of future obstetric complications, were also often not discussed during visits.

Our findings show suboptimal adherence to ANC standards and particularly large gaps for screening, counseling, and for certain preventive treatments such as deworming and calcium supplementation. We also found a high prevalence of concurrent diseases (anemia, malnutrition, chronic illnesses) and pregnancy risk factors (prior obstetric complications, multiple pregnancies, and being aged less than 20 or more than 35) among the women enrolled in the study. But women with these conditions did not appear to receive additional care. This suggests that women with risk factors were unlikely to receive targeted management to reduce the likelihood of future health problems, such as newborn low birth weight, eclampsia, or maternal and perinatal morbidity or mortality [6,7,18,37]. Similar to our findings, a study in 8 Eastern and Southern African countries found that twin pregnancies did not receive earlier, more frequent, or intensified ANC compared to singletons [7].

In regression analyses, some facility characteristics were associated with ANC completeness. This included better facility staffing in Ethiopia. In South Africa, better facility readiness was associated with higher ANC quality. This finding is not surprising given that the service readiness score included several equipment and supplies required for ANC such as blood pressure cuffs, scales, and blood and urine diagnostic tests. Lower patient volume was also associated with better ANC completeness in South Africa. Other studies have also shown that ANC quality is optimal in facilities with adequate staffing and equipment but with manageable workloads [38]. Ensuring sufficient time is spent with the pregnant woman is essential [38–40]. Surprisingly in India, better staffing was associated with lower quality ANC. This is likely due to residual confounding by facility size or type.

Multivariable regression models also showed that primipara women might receive lower quality ANC. This is particularly concerning given their greater risk for birth complications and the fact that poor knowledge of pregnancy complications is associated with an increased risk of maternal death [41–43]. In Ethiopia, Kenya, and South Africa, women initiated ANC late in pregnancy. This delay further hinders the health system’s ability to promptly detect and manage concurrent diseases and risks before the delivery. Delayed ANC is often multifactorial including both user and health system factors [44]. However, health systems should be alert to this key risk for poor outcomes and plan strategies to address it. Low pregnancy intent in South Africa also threatens the ability of the health system to ensure positive pregnancy outcomes and points to gaps in family planning services and contraceptive use [45,46].

We found that several risk factors with high prevalence in selected countries, such as malnutrition, chronic illnesses, or prior obstetric complications and danger signs were often overlooked during the first ANC visit and were poorly handled by health systems.

In Ethiopia and India, nearly one in 5 pregnant women in our sample was underweight (MUAC <23 cm or BMI <18.5). This is similar to the prevalence of undernutrition at national levels in both countries [47,48]. Pregnancy undernutrition accounts for a large proportion of premature birth and low birth weight, the leading causes of newborn mortality [5]. According to national guidelines in Ethiopia, a MUAC under 23 cm indicates acute malnutrition and is an indication for supplementation with ready-to-use foods such as *Plumpy’Nut*, or *corn-soy blend [CSB++]* until the measurement is in the normal range [24]. Nonetheless, only 5% to 6% of undernourished women in Ethiopia and India were given or prescribed food supplements. In both countries, food supplementation for pregnant women is generally provided through parallel programs such as Anganwadi in India. The government should consider integrating these efforts with routine ANC.

Ethiopia also had the lowest rate of calcium supplementation where less than 1% of women in their second or third trimester were given or prescribed calcium. This finding might be due to the introduction of calcium in the 2022 ANC guidelines in Ethiopia and may reflect a delay in adoption of the new guidelines. Nonetheless, Ethiopia also had the lowest rate of IFA supplementation. Only 70% of anemic women were given or prescribed IFA in Ethiopia (compared to 92% to 97% in the other 3 countries). Obesity is another well-known risk factor for poor pregnancy outcomes [33]. In our study, South Africa had the highest prevalence of women with a BMI ≥30 (32%) and the best ANC quality. However, only 42% of these women were counseled on nutrition and the importance of exercise during pregnancy.

Depression in pregnancy is increasingly acknowledged to be an important risk factor for a range of poor maternal and child outcomes [13–15,49]. For this reason, screening for depression is recommended by ANC guidelines in most countries, including Ethiopia, Kenya, and South Africa. Similar to other studies, we found a high prevalence of depressive symptoms among the pregnant women enrolled [49]. Yet, it was largely neglected by health systems. Few were screened for depression and only 11 women (<1%) across the 4 countries were referred to a mental health provider or prescribed antidepressants. Another striking finding is that several women with prior complications did not discuss their obstetric history with the provider during the visit. Similarly, not all previously diagnosed chronic illnesses were discussed and only 19% to 54% of women with self-reported danger signs received advice. This points to serious gaps in screening and history taking and to a clear inability of women to discuss important concerns during the visit.

A growing number of articles have explored the content and quality of ANC visits [2–4,6,7,22,23,38]. These have shown gaps in the provision of ANC components like blood and urine testing and counselling on pregnancy complications. However, there have been fewer studies investigating the detection and management of pregnancy risk factors during ANC in LMICs [7,6]. Unlike previous studies, we assessed the prevalence of several potential risk factors and the frequency of detection by health systems. Compared to previous studies with long recall periods, the eCohort also assessed ANC completeness immediately after the visit and included physical health assessments, and data from maternal health cards. Despite these strengths, our study has several limitations. First, most of the indicators on the content of ANC are based on self-reports and more educated or older women might better recall or understand the different clinical actions that took place and the provider’s instructions. Although this is a limitation, it might also point to the need for more tailored provider communication to address the needs of adolescents or less educated women. Second, the BMI was measured during the first ANC visit which took place at any point during the pregnancy. Therefore, BMI estimates for women already in their second or third trimester may be overestimated. This means that undernutrition based on pregnancy BMI may be underestimated while obesity is overestimated. The pregnancy danger signs (e.g., severe headaches, severe abdominal pain) were self-reported and may not indicate true pregnancy danger signs. However, these complaints should be investigated by providers during the visit. The 4 countries also included a different mix of facility types. In India and South Africa, we were unable to recruit women in private health facilities. Therefore, these findings may not be representative of the entire sites selected, but only reflect public sector care. Similarly, our study is not nationally representative but reflects the levels of ANC quality in the sites selected and in the public sectors of the Indian and South African sites. In our analyses, we also lacked information on the ANC providers. Their training, years of experience, or other individual factors may affect the quality of ANC. Our measures for the detection and correct management of risk factors are limited as these data are largely based on patient self-reports. Specifically, we lack information on the reasons why some women were referred to hospitals for follow-up care. In India, referral to hospital is done routinely, even among “low risk” women, to ensure that women can see a specialist at least once during their pregnancy. In the case of nutritional assessments, measuring weight and height does not always mean that the provider correctly calculates and interprets the BMI. Finally, the findings in this paper relate only to the first ANC visit. Future analyses of eCohort data will assess whether follow-up visits resulted in better detection and management of health problems in pregnancy and whether ANC completeness is associated with birth outcomes.

In 2016, the WHO released new ANC guidelines that recommend a minimum of 8 ANC visits instead of 4 to improve the likelihood of detecting and addressing pregnancy risks and concurrent illnesses. Our analysis shows poor levels of detection in the very first ANC visit and a lack of adherence to ANC standards. In this context, simply increasing the number of ANC visits is unlikely to improve health outcomes. Our findings highlight the need for better assessment, communication, and referral of pregnant women between the primary and secondary levels of the health system. Data from the eCohort can immediately guide regional health managers to develop improvement strategies to improve adherence to standards. It should also prompt national policymakers to interrogate structural factors, such as preservice training, supervision, management norms, facility infrastructure, data systems, and continuing education, that ultimately influence performance at the point of care.

**Box 1. Clinical actions included in ANC completeness scores and required in the first ANC visit according to national guidelines in 4 countries**

**Physical examinations**

Blood pressure measurement

Weight measurement

Height measurement ^a^

Mid-upper arm circumference measurement ^a^

**Diagnostic tests**

Blood sample collected (blood draw or finger prick)

Urine sample collected

Ultrasound conducted (third trimester only) ^b^

**History taking and screening**

Date of last menstrual period assessed

Depression screening ^a^

Danger sign screening ^a, c^

Previous pregnancies discussed

**Counselling**

Nutrition

Exercise ^a^

Signs of pregnancy complications

Birth preparedness

Estimated due date given (second or third trimester only)

Told to return for additional antenatal care

**Treatments and prevention**

Iron and folic acid pills (given or prescribed)

Calcium supplements (given or prescribed, second or third trimester only) ^c^

Deworming medication (given or prescribed, second or third trimester only) ^b^

Tetanus toixoid vaccination (given to women not already protected)

Insecticide treated bed net (given or prescribed, in malaria endemic sites only) ^a, b^

All 22 items are required according to Ethiopian national antenatal care guidelines. In Kenya, 20 items were included in the ANC completeness score, while 19 items were included in South Africa, and 16 items in India.

- Not included in India.
- Not included in South Africa.
- Not included in Kenya.

## Supporting information

S1 Information TABLE A in S1 Information. CHARACTERISTICS OF THE 8 STUDY SITES SELECTED IN 4 COUNTRIES. TABLE B in S1 Information. RECOMMENDED CONTENT OF ANTENATAL CARE BASED ON NATIONAL GUIDELINES IN ETHIOPIA, SOUTH AFRICA, INDIA, AND KENYA. TABLE C in S1 Information. QUESTIONS USED TO ASSESS HEALTH LITERACY. ANALYSIS PLAN: THE MATERNAL AND NEWBORN HEALTH (MNH) ECOHORT TO TRACK LONGITUDINAL CARE QUALITY. TABLE D in S1 Information. CHARACTERISTICS OF 94 HEALTH FACILITIES INCLUDED IN THE ANALYSIS. TABLE E in S1 Information. CATEGORIES OF SERVICE READINESS, STAFFING, AND ANC VOLUMES USED IN REGRESSION ANALYSES BY COUNTRY. TABLE F in S1 Information. CONTENT OF 4,068 FIRST ANTENATAL CARE VISITS IN 4 COUNTRIES. TABLE G in S1 Information. CHARACTERISTICS OF WOMEN ACCORDING TO PRENATAL RISK PROFILE. TABLE H in S1 Information. RESULTS OF LINEAR MULTILEVEL REGRESSION MODELS FOR THE COMPLETENESS OF THE FIRST ANTENATAL CARE VISIT IN 4 COUNTRIES. TABLE E in S1 Information. STROBE CHECKLIST.
(DOCX)

## Abbreviations

ANC: antenatal care
BMI: body mass index
CI: confidence interval
Hb: hemoglobin
IFA: iron and folic acid
LMIC: low- and middle-income country
MNH: maternal and newborn health
MUAC: mid-upper arm circumference
PHQ9: Patient Health Questionnaire– 9
SARA: service availability and readiness assessment
WHO: World Health Organization
