# Supplementary material for: Antenatal care quality and detection of risk among pregnant women: An observational study in Ethiopia, India, Kenya, and South Africa
Source: PLoS Med. 2024 Aug 27;21(8):e1004446. doi: 10.1371/journal.pmed.1004446 (PMC11349107; doi:10.1371/journal.pmed.1004446)
Supplement: S1 Information — TABLE A in S1 Information. CHARACTERISTICS OF THE 8 STUDY SITES SELECTED IN 4 COUNTRIES. TABLE B in S1 Information. RECOMMENDED CONTENT OF ANTENATAL CARE BASED ON NATIONAL GUIDELINES IN ETHIOPIA, SOUTH AFRICA, INDIA, AND KENYA. TABLE C in S1 Information. QUESTIONS USED TO ASSESS HEALTH LITERACY. ANALYSIS PLAN: THE MATERNAL AND NEWBORN HEALTH (MNH) ECOHORT TO TRACK LONGITUDINAL CARE QUALITY. TABLE D in S1 Information. CHARACTERISTICS OF 94 HEALTH FACILITIES INCLUDED IN THE ANALYSIS. TABLE E in S1 Information. CATEGORIES OF SERVICE READINESS, STAFFING, AND ANC VOLUMES USED IN REGRESSION ANALYSES BY COUNTRY. TABLE F in S1 Information. CONTENT OF 4,068 FIRST ANTENATAL CARE VISITS IN 4 COUNTRIES. TABLE G in S1 Information. CHARACTERISTICS OF WOMEN ACCORDING TO PRENATAL RISK PROFILE. TABLE H in S1 Information. RESULTS OF LINEAR MULTILEVEL REGRESSION MODELS FOR THE COMPLETENESS OF THE FIRST ANTENATAL CARE VISIT IN 4 COUNTRIES. TABLE E in S1 Information. STROBE CHECKLIST. (DOCX) [file pmed.1004446.s001.docx]

**Antenatal care quality and detection of risk among pregnant women: an observational study in Ethiopia, India, Kenya, and South Africa**

**Supplemental materials**

[Supplemental Table A. Characteristics of the eight study sites selected in four countries 2](#_Toc172727667)

[Supplemental Table B. Recommended content of antenatal care based on national guidelines in Ethiopia, South Africa, India, and Kenya 3](#_Toc172727668)

[Supplemental table C. Questions used to assess health literacy 5](#_Toc172727669)

[Protocol and statistical analysis plan: the maternal and newborn health (MNH) eCohort to track longitudinal care quality 6](#_Toc172727670)

[Supplemental table D. Characteristics of 94 health facilities included in the analysis 8](#_Toc172727671)

[Supplemental table E. Categories of service readiness, staffing and ANC volumes used in regression analyses by country 9](#_Toc172727672)

[Supplemental Table F. Content of 4,068 first antenatal care visits in four countries 10](#_Toc172727673)

[Supplemental table G. Characteristics of women according to prenatal risk profile 12](#_Toc172727674)

[Supplemental table H. Results of linear multi-level regression models for the completeness of the first antenatal care visit in four countries 13](#_Toc172727675)

[Supplemental Table E. STROBE Statement 15](#_Toc172727676)

## **Supplemental Table A. Characteristics of the eight study sites selected in four countries**

|  | **Ethiopia** | | **India** | | **Kenya** | | **South Africa** | |
| --- | --- | --- | --- | --- | --- | --- | --- | --- |
| Baseline survey dates | April-May 2023 | | November 2023-January 2024 | | June 2023-September 2023 | | May 2023-October 2023 | |
| Languages | Amharic, Afan, Oromo | | Hindi | | Kiswahili, Kamba, English | | IsiZulu, English | |
| Study sites | Adama Town | East Shewa | Jodhpur | Sonipat | Kitui | Kiambu | Nongoma | Umhlathuze |
| Administrative level | Zones in Oromia Region | | District, State of Rajasthan | District, State of Haryana | County in the former Eastern Province | County in the former Central Province (located near the capital Nairobi) | Local municipality, Zululand district, Province of KwaZulu-Natal | Local municipality, King Cetshwayo District, Province of KwaZulu-Natal |
| Total Population | 222,035 | 1,357,522 | 4,720,000 | 1,640,000 | 1,136,187 | 2,417,735 | 212,000 | 450,000 |
| Area (km^2^) | 29.86 | 8,371 | 22,850 | 2,122 | 30,496 | 2,544 | 2,182 | 1,233 |
| Population density (people per km^2^) | 7,435 | 162 | 207 | 773 | 37 | 981 | 103 | 334 |
| Rurality / Classification | Urban | Rural | 34.3% urban  65.7% rural | 31.3% urban  68.7% rural | Rural (Arid and Semi-Arid Land (ASAL)) | Urban | Rural | Urban |
| **Types of facilities selected for recruitment of pregnant women in the eCohort:** | | | | | | | | |
|  | **Ethiopia** | | **India** | | **Kenya** | | **South Africa** | |
| Public primary | Public health centers | | Primary Health Centers and subcenters | | Government dispensaries and health centers. | | Primary care clinics | |
| Private primary | Private clinics and MCH centers | | N/A | | Private and faith-based clinics, dispensaries, and health centers | | N/A | |
| Public secondary | Primary and general hospitals | | District hospitals and Community Health Centers | | Sub district hospital, Sub County hospital, County referral hospital | | N/A | |
| Private secondary | Private hospitals | | N/A | | Faith-based and private hospitals | | N/A | |

## **Supplemental Table B. Recommended content of antenatal care based on national guidelines in Ethiopia, South Africa, India, and Kenya**

|  | | **Ethiopia** | **Kenya** | **South Africa** | **India** |
| --- | --- | --- | --- | --- | --- |
| **Physical examinations** | General appearance | ● | ● | ● | ● |
|  | Jaundice | ● |  | ● | ● |
|  | Edema | ● |  | ● | ● |
|  | Blood pressure | ● | ● | ● | ● |
|  | Heart rate | ● | ● | ● | ● |
|  | Respiratory rate | ● | ● | ● | ● |
|  | Temperature | ● |  | ● |  |
|  | Weight | ● | ● | ● | ● |
|  | Height | ● | ● | ● |  |
|  | MUAC | ● | ● | ● |  |
|  | Mucous membrane | ● | ⁎ | ● | ● |
|  | Chest | ● | ⁎ | ● | ● |
|  | Heart | ● | ⁎ | ● | ● |
|  | Breast |  | ● | ● | ● |
|  | Fundal height | ● | ● | ● | ● |
|  | Fetal presentation | ● | ● | ● | ● |
|  | Fetal heartbeat | ● | ● | ● | ● |
|  | Fetal movement | ● | ● | ● | ● |
|  | Abdomen palpation | ● | ● | ● | ● |
|  | Signs of trauma | ● |  |  |  |
|  | Vaginal examination | ● ^a^ | ● | ● | ● |
|  | Examine FGM scar | ● | ● |  |  |
|  | Dental health |  |  | ● |  |
| **Blood test** | Hemoglobin or hematocrit | ● | ● | ● | ● |
|  | Blood group | ● | ● | ● (by indication) | ● |
|  | Rh | ● | ● | ● | ● |
|  | Thyroid stimulating hormone |  |  |  | ● (by indication) |
| **Urine analysis** | Urine dipstick | ● | ● | ● | ● |
|  | Microscopy | ● | ● |  | ● |
|  | Gram stain | ● | ● |  | ● |
| **Other tests** | HIV | ● | ● | ● | ● |
|  | HBV | ● | ● |  | ● |
|  | Syphilis | ● | ● | ● | ● |
| **Ultrasound and other screening** | Ultrasound | ● | ● | ● (by indication) ^e^ | ● |
|  | GDM screening | ● (by indication) ^b^ | ● | ● (by indication) ^f^ | ● |
|  | TB screening | ● (by indication) ^c^ | ● | ● |  |
|  | GBS screening | ● (by indication) ^d^ |  |  |  |
|  | Malaria test | ● (endemic areas) | ● (endemic areas) | ● (endemic areas) | ● (endemic areas) |
| **History taking** | Menstrual history | ● | ● | ● | ● |
|  | Present pregnancy | ● | ⁎ | ● | ● |
|  | Pregnancy intention | ● | ⁎ |  | ● |
|  | Past obstetric history | ● | ● | ● | ● |
|  | Medical history | ● | ● | ● | ● |
|  | Current medication | ● | ⁎ | ● | ● |
|  | Gynecologic history | ● | ⁎ |  |  |
|  | Nutritional history | ● | ⁎ |  |  |
|  | Social and personal history | ● | ⁎ | ● | ● |
|  | Depression | ● | ● | ● |  |
|  | Intimate partner violence | ● | ⁎ |  | ● |
|  | Family history | ● | ⁎ | ● | ● |
| **Risk identification** | Risk identification and stratification | ● | ● | ● | ● |
| **Counseling on the following** | Nutrition | ● | ● | ● | ● |
|  | Substance use | ● | ● | ● | ● |
|  | Exercise | ● | ● | ● |  |
|  | Sexual activity | ● |  | ● | ● |
|  | Hygiene and sanitation | ● | ● | ● |  |
|  | Dental hygiene | ● | ● |  |  |
|  | Sleep and rest | ● | ● |  | ● |
|  | Danger signs | ● | ● | ● | ● |
|  | Birth preparedness | ● | ● | ● | ● |
|  | Family planning | ● | ● | ● | ● |
|  | Domestic violence | ● | ● | ● | ● |
|  | ITN use | ● | ● |  | ● |
| **Nutritional interventions** | Provide iron and folic acid | ● | ● | ● | ● |
|  | Provide Calcium | ● |  | ● | ● |
| **Preventative intervention** | Give tetanus toxoid vaccination | ● | ● | ● | ● |
|  | Deworming medication | ● | ● |  | ● |
|  | Prevent and manage RhD isoimmunization | ● | ● | ● | ● |
|  | Provide low dose aspirin | ● | ● | ● | ● |
|  | Insecticide treated bed nets | ● (endemic areas) | ● (endemic areas) | ● (endemic areas) | ● (endemic areas) |
| **Recommended total number of ANC visits** | | 8 | 8 | 5 | 4 |
| **Timing of ANC visits according to gestational age in weeks** | | Up to 12, 20, 26, 30, 34, 36, 38, 40 | 8-12, 20, 26, 30, 34, 36, 38, 40 | <12, 20, 26-28, 32-34, 38 (41 if still pregnant) | 12, 16-26, 28-34, 36 to term |

● recommended according to national guidelines

⁎ recommended in broad terms

^a^ Vaginal examination is not a routine practice during ANC. The most common indications are for symptoms of STI, history of FGM, screening for precancerous cervical lesion, vaginal bleeding, suspected leakage of amniotic fluid, and suspected preterm labor.

^b^ Indicated when there is personal or family history, previous macrosomia or stillbirth, obese, large-for date uterus, family history, glycosuria

^c^ Indicated when there is current cough, weight loss/failure to gain weight, night sweats, and fever.

^d^ Indicated when there was previous perinatal infection with Group B streptococcus

^e^ An ultrasound scan for gestational age estimation should be requested for women who are unsure of dates with SFH measurement less than 24 cm.

^f^ All pregnant women with risk factors for diabetes in pregnancy should be screened at the first antenatal visit and again at 28 weeks gestation if the initial screen was negative.

The below documents were reviewed to construct the ANC completeness indices:

**Ethiopia:**

1. Ministry of Health Ethiopia, National Antenatal Care Guidelines. 2022.
2. Federal Ministry of Health, Integrated Antenatal, Labor, Delivery, Newborn and Postnatal Care Card.

**Kenya:**

1. Ministry of Health Republic of Kenya, National Guidelines on Quality Obstetrics and Perinatal Care 2020.
2. Ministry of Health Republic of Kenya, Mother and Child Handbook. 2020. Available from: <https://familyhealth.go.ke/wp-content/uploads/2020/11/Mother-Child-Health-Handbook-MOH-September-2020.pdf>

**South Africa:**

1. Department of Health Republic of South Africa, Guidelines for Maternity Care in South Africa. A manual for clinics, community health centers and district hospitals. 2016. Available from <https://knowledgehub.health.gov.za/elibrary/guidelines-maternity-care-south-africa-2016>
2. Department of Health Republic of South Africa, Maternity Case Record. Described in: Guidelines for Maternity care in South Africa. A manual for clinics, community health centers and district hospitals. 2016.

**India:**

1. Ministry of Health and Family Welfare of India, Guidelines for Antenatal Care and Skilled Attendance at Birth by ANMs/LHVs/SNs. 2010. Available from [https://nhmodisha.gov.in/writereaddata/Upload/Guidelines/Guidelines%20for%20ANC%20and%20SAB%20by%20ANMs,LHVs,SNs.pdf](https://nhmodisha.gov.in/writereaddata/Upload/Guidelines/Guidelines%20for%20ANC%20and%20SAB%20by%20ANMs,LHVs,SNs.pdf%20)
2. Ministry of Health and Family Welfare India, Guidelines for Pregnancy Care and Management of Common Obstetric Complications by Medical Officers. 2005. Available from <https://nhmodisha.gov.in/writereaddata/Upload/Documents/Normal_delivery_and_management_of_obstetric_complications_.pdf>
3. Ministry of Health and Family Welfare of India and Ministry of Women and Child Development India, Mother and Child Protection Card. 2018. Available from <https://nhm.gov.in/New_Updates_2018/NHM_Components/Immunization/Guildelines_for_immunization/MCP_Card_English_version.pdf>

## **Supplemental table C. Questions used to assess health literacy**

| **1** | Now I would like to talk about something else. Have you ever heard of an illness called HIV/AIDS? | 1 Yes  0 No 🡪 GO TO 3 |
| --- | --- | --- |
| **2** | Do you think that people can get the HIV virus from mosquito bites? | 1 Yes  0 No  99 Don’t know |
| **3** | Have you ever heard of an illness called tuberculosis or TB? | 1 Yes  0 No 🡪 GO TO 5 |
| **4** | Do you think that TB can be treated using herbal or traditional medicine made from plants? | 1 Yes  0 No  99 Don’t know |
| **5** | When children have diarrhea, do you think that they should be given less to drink than usual, more to drink than usual, about the same or it doesn’t matter? | 1 Less than usual  2 More than usual  3 About the same  4 It doesn’t matter  99 Don’t know |
| **6** | Is smoke from a wood burning traditional stove good for health, harmful for health or do you think it doesn’t really matter? | 1 Good  2 Harmful  3 Doesn’t matter |

Adapted from the India Health and Human Development Survey (IHDS): Desai, S. and Vanneman, R., 2015. *India human development survey-ii (ihds-ii), 2011-12* (Vol. 31). Ann Arbor, MI: Inter-university Consortium for Political and Social Research.

## **Protocol and statistical analysis plan: the maternal and newborn health (MNH) eCohort to track longitudinal care quality**

**Aim and study design**

The MNH eCohort is a longitudinal mixed mode (in-person and phone) survey aimed at collecting near-real time data on health system quality in selected sites. The MNH eCohort has four goals: (1) to measure system competence across the continuum of MNH care, (2) to describe health outcomes, user experience, and care pathways in the health system for women and newborns, (3) to identify gaps in effective care provision for good health outcomes, and (4) to build a flexible measurement tool for assessment of health system performance using mobile phones.

**Sample and respondent selection**

Two sentinel sites will be selected in each implementing country (one predominantly rural, and one predominantly urban). Our target population will be pregnant women who use ANC services in each site (e.g., a zone in Ethiopia, county in Kenya, district municipality in South Africa and a district in India). Recruitment will be conducted in person at health facilities while women attend their first ANC consultation. Women will be recruited in health facility types that are representative of care seeking patterns in the site. For example, we will use data from the health management information system to determine the proportion of women who attend their first ANC visit in public primary care facilities, public hospitals and private clinics or hospitals. The facilities selected for enrollment will be selected within these strata by probability proportional to size (using ANC patient volume as a measure of size). We aim to recruit a minimum of 50 women per facility strata in each site. There will be no restriction on the gestational age at enrollment (some women may seek ANC in their first trimester, while others may be enrolled late in their third trimester). Other inclusion criteria include being aged at least 15 years old, being at the facility to receive the first ANC visit and planning to continue to reside in the study site. We aim for 500 pregnant women to be recruited in each site (1000 per country).

**Data collection procedures**

The eCohort consists of five women survey modules. Module 1, the baseline survey, will be administered in person at enrollment in the health facility where women receive their first ANC visit. The baseline survey will be administered using tablets and we aim for enrollment to take no longer than 2 months. Data collectors will then contact women by phone every month (through computer-assisted-telephone interviewing) to administer the prenatal phone survey module (module 2). During these monthly calls, the data collector will determine whether women are still pregnant or whether they have given birth. Once she has delivered, module 3 will be administered to collect information on delivery care and outcomes. For consistency across respondents, they will administer the third module 2 to 4 weeks after the delivery. One month later, a phone survey on PNC care and outcomes will be administered (module 4, 6-8 weeks postpartum). Finally, an endline in-person survey will be conducted at 10-12 weeks postpartum. Follow-up time will vary depending on the stage of pregnancy at enrollment. The MNH eCohort is primarily based on self-reports but we aim to complement self-reported information with data from two additional sources: a brief health facility survey and physical health assessments. A short health facility assessment (module 0) will allow us to describe the structural quality and size of health facilities where women are recruited. The baseline and endline in-person surveys will also include brief physical assessments (women’s weight, height, blood pressure, mid-upper arm circumference, hemoglobin level and baby’s weight, length, and head circumference).

Respondents will be compensated for their participation. At recruitment women will be offered a mobile phone (to ensure they can do the phone surveys). Those who already own a phone will be offered the equivalent monetary value in airtime. During the baseline survey, data collectors will determine the preferred phone number to use for follow-up and they will collect a list of backup phone numbers to facilitate call tracing (e.g., phone numbers of family members or friends). Respondents will also receive airtime after completion of each monthly phone survey as well as compensation for participating in the in-person endline survey (e.g., a changing pad and diapers).

**Statistical methods**

The first paper will assess care and system competence during the first ANC visit in four countries. The paper will aim to assess whether women with risk factors receive additional care and targeted management of their conditions during the first ANC visit. We will use descriptive analyses to describe demographic characteristics of participants, their health status, and the quality of the first antenatal care visit as well as characteristics of health facilities where women receive antenatal care. For women with risk factors (e.g. chronic illnesses, prior obstetric complications, undernutrition, etc.), we will also assess whether the health providers have screened the women for the specific health problems and whether the health condition is addressed during the first ANC visit. In descriptive analyses, categorial variables will be presented as percentages and means, and standard deviations will be presented for continuous variables.

We will use linear regression analyses to assess the factors associated with ANC quality. An alpha level of 0.05 will indicate statistical significance. We will use a complete case analysis method if missing data is below 10% for key variables of interest. Analyses will be conducted in each country separately and standard errors will be adjusted for clustering by health facilities. Before performing the multiple regression analyses, we will confirm the absence of multicollinearity among the study covariates using variance inflation factors.

Key variables of interest will include:

*Dependent variables:*

Quality of the first ANC visit. We will create quality scores that measure clinical performance of recommended clinical actions during the first ANC visit (e.g. blood pressure measurement, blood test, ultrasound, depression screening iron and folic acid supplements etc.). Quality scores will be based on national recommendations for ANC and will be country specific.

*Independent variables:*

The main predictor of interest will be the women’s risk profile. We will select a series of health indicators that may indicate women at risk of poor pregnancy outcomes. These will be selected based on previous literature and may include maternal age, concurrent chronic illnesses, multiple pregnancies, nutritional status etc. Independent variables will also include key demographic factors that may influence the quality of ANC such as wealth, education, and marital status.

Analyses will be conducted using STATA or R and according to principles or reproducible research. Statistical code will be shared through a GitHub repository for version control.

## **Supplemental table D. Characteristics of 94 health facilities included in the analysis**

|  |  |  | **Public facilities** | **Private facilities** | **Primary care facilities** | **Secondary facilities (hospitals)** | **Service readiness index score ^a^** | **Total number of staff providing obstetric care** | **Average number of ANC visits provided per month ^b^** |
| --- | --- | --- | --- | --- | --- | --- | --- | --- | --- |
|  | **Site** | **N** | **N (%)** | **N (%)** | **N (%)** | **N (%)** | **mean (SD)** | **mean (SD)** | **mean (SD)** |
| **Ethiopia** | Rural | 11 | 10 (90.9%) | 1 (9.1%) | 10 (90.9%) | 1 (9.1%) | 0.57 (0.16) | 2.45 (0.93) | 135.91 (69.70) |
|  | Urban | 10 | 5 (50.0%) | 5 (50.0%) | 7 (70.0%) | 3 (30.0%) | 0.71 (0.16) | 5.40 (3.84) | 180.10 (85.24) |
|  |  | **21** |  |  |  |  |  |  |  |
| **Kenya** | Rural | 12 | 8 (66.7%) | 4 (33.3%) | 8 (66.7%) | 4 (33.3%) | 0.80 (0.13) | 15.67 (13.08) | 162.81 (175.21) |
|  | Urban | 9 | 5 (55.6%) | 4 (44.4%) | 5 (55.6%) | 4 (44.4%) | 0.88 (0.09) | 30.78 (20.30) | 415.70 (269.23) |
|  |  | **21** |  |  |  |  |  |  |  |
| **India** | Rural | 15 | 15 (100.0%) | N/A | 12 (80.0%) | 3 (20.0%) | 0.59 (0.23) | 18.87 (13.42) | 85.94 (227.41) |
|  | Urban | 14 | 14 (100.0%) | N/A | 9 (64.3%) | 5 (35.7%) | 0.64 (0.16) | 21.57 (32.42) | 70.21 (35.45) |
|  |  | **29** |  |  |  |  |  |  |  |
| **South Africa** | Rural | 9 | 9 (100.0%) | N/A | 9 (100.0%) | N/A | 0.80 (0.05) | 11.22 (7.82) | 158.06 (188.23) |
|  | Urban | 13 | 13 (100.0%) | N/A | 13 (100.0%) | N/A | 0.77 (0.07) | 8.31 (9.55) | 301.90 (224.26) |
|  |  | **22** |  |  |  |  |  |  |  |

a. The service readiness score included three dimensions of structural quality: presence of basic amenities, availability and functionality of basic equipment required for maternal and newborn health care, and diagnostic capacity. Detailed indicators are shown below.

b. Based on antenatal care volumes in the past year.

| **The service readiness score is the average of the below three scores:** | |
| --- | --- |
| **Basic amenities** | Presence of: improved water, improved sanitation, electricity, telephone, computer, internet, and ambulance. |
| **Basic equipment for maternal and newborn health care** | At least one functional in the facility at the time of the survey: blood pressure cuff, adult scale, infant scale, thermometer, stethoscope, and ultrasound. |
| **Diagnostic capacity** | At least one available and valid at the time of the survey: malaria rapid diagnostic kit, syphilis rapid test kit, HIV rapid test kit, urine pregnancy test kit, dipsticks for urine protein, dipsticks for urine glucose, dipsticks for urine ketone bodies.  The following is performed onsite: blood glucose tests using a glucometer, haemoglobin testing, general microscopy/wet-mounts, HIV antibody testing by ELISA. |

## **Supplemental table E. Categories of service readiness, staffing and ANC volumes used in regression analyses by country**

|  | **Ethiopia** | **Kenya** | **India** | **South Africa** |
| --- | --- | --- | --- | --- |
| **Service readiness score** |  |  |  |  |
| **Low** | 0.28-0.51 | 0.58-74 | 0.17-0.48 | 0.67-0.74 |
| **Middle** | 0.53-0.69 | 0.77-0.85 | 0.60-0.72 | 0.77-0.83 |
| **High** | 0.72-0.97 | 0.88-0.97 | 0.75-0.88 | 0.83-0.88 |
| **Number of staff providing obstetric care** |  |  |  |  |
| **Low** | 1-2 | 4-9 | 5-11 | 1-4 |
| **Middle** | 3 | 11-24 | 12-18 | 5-19 |
| **High** | 4-14 | 28-66 | 20-133 | 20-28 |
| **Average number of ANC visits per month** |  |  |  |  |
| **Low** | 37-146 | 33-154 | 7-24 | 20-149 |
| **Middle** | 147-202 | 157-498 | 30-65 | 168-465 |
| **High** | 230-305 | 550-936 | 78-902 | 480-776 |

## **Supplemental Table F. Content of 4,068 first antenatal care visits in four countries**

|  | **Ethiopia** | | **Kenya** | | **India** | | **South Africa** | |
| --- | --- | --- | --- | --- | --- | --- | --- | --- |
|  | **East Shewa  N=508** | **Adama Town  N=492** | **Kitui N=504** | **Kiambu  N=498** | **Rural**  **N=365** | **Urban**  **N=657** | **Nongoma  N=516** | **uMhlathuze  N=528** |
| **Physical examinations** |  |  |  |  |  |  |  |  |
| Blood pressure | 56.5% | 90.0% | 93.5% | 98.6% | 99.5% | 98.2% | 99.6% | 100.0% |
| Weight | 72.2% | 92.7% | 88.7% | 99.4% | 99.7% | 98.5% | 100.0% | 99.8% |
| Height | 2.6% | 3.1% | 79.0% | 58.7% |  |  | 99.0% | 99.8% |
| MUAC | 18.7% | 3.3% | 22.7% | 24.7% |  |  | 90.7% | 98.5% |
| **Diagnostic tests** |  |  |  |  |  |  |  |  |
| Blood draw or blood finger prick | 91.1% | 99.0% | 97.0% | 98.8% | 81.4% | 92.7% | 100.0% | 99.8% |
| Urine test | 72.8% | 99.0% | 86.9% | 92.6% | 65.7% | 67.7% | 99.6% | 99.4% |
| Ultrasound (3^rd^ trimester) ^a^ | 27.6% | 74.2% | 6.8% | 16.0% | 18.8% | 48.6% |  |  |
| **History taking and screening** |  |  |  |  |  |  |  |  |
| Assessed date of last menstrual period | 69.8% | 86.6% | 99.8% | 98.8% | 98.4% | 97.1% | 97.5% | 96.4% |
| Screened for depression | 3.0% | 4.5% | 19.9% | 32.1% |  |  | 21.2% | 7.8% |
| Screened for danger signs ^b^ | 16.1% | 28.0% |  |  |  | | 16.5% | 48.4% |
| Discussed previous pregnancies ^c^ | 41.3% | 50.8% | 61.9% | 59.1% | 69.9% | 79.6% | 84.2% | 84.6% |
| **Counselling** |  |  |  |  |  |  |  |  |
| Counseled on nutrition | 29.4% | 37.3% | 50.2% | 68.2% | 86.3% | 63.6% | 46.3% | 61.3% |
| Counseled on exercise | 3.6% | 8.0% | 32.9% | 52.8% |  |  | 35.9% | 53.0% |
| Counseled on signs of pregnancy complications | 22.2% | 40.7% | 45.9% | 75.3% | 8.0% | 18.6% | 50.6% | 48.0% |
| Counseled on birth preparedness | 8.9% | 6.7% | 22.6% | 54.0% | 61.7% | 51.5% | 43.3% | 51.7% |
| Given an estimated due date (≥2^nd^ trimester) ^c^ | 0.8% | 2.5% | 72.4% | 81.5% | 61.7% | 69.9% | 63.4% | 78.8% |
| Told to return for second ANC visit | 85.2% | 94.3% | 94.2% | 98.0% | 92.6% | 71.9% | 97.3% | 89.1% |
| **Treatments and prevention** |  |  |  |  |  |  |  |  |
| Given or prescribed iron and folic acid pills | 79.3% | 81.1% | 90.3% | 95.6% | 95.3% | 90.2% | 86.5% | 98.9% |
| Given or prescribed calcium supplements (≥2^nd^ trimester) ^d^ | 0.9% | 0.3% |  |  | 71.9% | 71.8% | 81.1% | 80.4% |
| Given or prescribed deworming medication (≥2^nd^ trimester) ^d^ | 0.5% | 0.0% | 12.6% | 4.0% | 6.7% | 18.6% |  |  |
| Given a dose of tetanus toxoid vaccine ^e^ | 79.9% | 75.2% | 82.7% | 52.4% | 60.2% | 62.3% | 92.4% | 95.3% |
| Given or prescribed insecticide treated bed net ^f^ | 0.0% | 1.5% | 90.9% | 33.7% |  |  |  |  |

a. Only among women already in third trimester of pregnancy at first antenatal care visit.

b. Due to data collection error, danger sign screening was not measured in Kenya and India despite being recommended as per national guidelines.

c. Among multiparous women.

d. Only among women already in second or third trimester of pregnancy at first ANC visit.

e. Only among women not already protected against tetanus (2 or more doses (the last one within 3 years), 3 or more doses (the last one within 5 years), 4 or more doses (the last one within 10 years) or 5 or more lifetime doses).

f. Only in sites where malaria is endemic and among women who did not already have an insecticide treated bed net.

## **Supplemental table G. Characteristics of women according to prenatal risk profile**

|  | **Ethiopia** | | **Kenya** | | **India** | |
| --- | --- | --- | --- | --- | --- | --- |
|  | **Rural** | **Urban** | **Rural** | **Urban** | **Rural** | **Urban** |
|  | **N=508** | **N=492** | **N=504** | **N=498** | **N=365** | **N=657** |
|  |  |  |  |  |  |  |
|  | **N (%)** | **N (%)** | **N (%)** | **N (%)** | **N (%)** | **N (%)** |
| No risk factor | 213 (41.9%) | 221 (44.9%) | 132 (26.2%) | 187 (37.6%) | 110 (30.1%) | 207 (31.5%) |
| One risk factor | 200 (39.4%) | 201 (40.9%) | 223 (44.2%) | 191 (38.4%) | 169 (46.3%) | 272 (41.4%) |
| Two or more risk factors | 95 (18.7%) | 70 (14.2%) | 149 (29.6%) | 120 (24.1%) | 86 (23.6%) | 178 (27.1%) |

|  | **South Africa** | |
| --- | --- | --- |
|  | **Rural** | **Urban** |
|  | **N=516** | **N=528** |
|  |  |  |
|  | **N (%)** | **N (%)** |
| No risk factor | 75 (14.5%) | 83 (15.7%) |
| One risk factor | 183 (35.5%) | 181 (34.3%) |
| Two risk factors | 153 (29.7%) | 171 (32.4%) |
| Three or more risk factors | 105 (20.3%) | 93 (17.6%) |

Categories of risk are the count of risk factors out of eight potential risk factor or group of risk factors: anemia (Hb<11 g/dL), chronic illness, underweight, obesity, prior obstetric complication, known multiple pregnancy, age > 35 or age < 20.

## **Supplemental table H. Results of linear multi-level regression models for the completeness of the first antenatal care visit in four countries**

|  |  | **Ethiopia** | | | | **Kenya** | | | | **India** | | | |
| --- | --- | --- | --- | --- | --- | --- | --- | --- | --- | --- | --- | --- | --- |
|  |  | **Coeff** | **LCL** | **UCL** | **p-value** | **Coeff** | **LCL** | **UCL** | **p-value** | **Coeff** | **LCL** | **UCL** | **p-value** |
| **Risk factors** | |  |  |  |  |  |  |  |  |  |  |  |  |
| No risk factor | | *ref.* |  |  |  | *ref.* |  |  |  | *ref.* |  |  |  |
| One risk factor | | 0.71 | -0.55 | 1.97 | 0.270 | 1.64 | 0.32 | 2.96 | 0.015 | 0.12 | -1.55 | 1.79 | 0.888 |
| Two or more risk factors | | 1.23 | -1.40 | 3.85 | 0.359 | 1.68 | -0.12 | 3.48 | 0.067 | 1.72 | -0.03 | 3.47 | 0.054 |
| **Demographics** | |  |  |  |  |  |  |  |  |  |  |  |  |
| Age |  |  |  |  |  |  |  |  |  |  |  |  |  |
|  | 20-34 | *ref.* |  |  |  | *ref.* |  |  |  | *ref.* |  |  |  |
|  | < 20 | 0.04 | -2.81 | 2.90 | 0.976 | -2.07 | -4.64 | 0.50 | 0.114 | -1.45 | -3.28 | 0.37 | 0.118 |
|  | 35+ | -2.21 | -5.31 | 0.89 | 0.162 | 0.39 | -0.91 | 1.69 | 0.557 | -2.86 | -6.88 | 1.17 | 0.164 |
| Completed secondary school | | 0.57 | -1.34 | 2.48 | 0.555 | 1.57 | 0.26 | 2.89 | 0.019 | -1.34 | -2.44 | -0.24 | 0.017 |
| Answers health literacy questions correctly | | 0.57 | -1.28 | 2.43 | 0.544 |  |  |  |  | 0.84 | -0.96 | 2.63 | 0.360 |
| Wealth |  |  |  |  |  |  |  |  |  |  |  |  |  |
|  | Poorest | *ref.* |  |  |  | *ref.* |  |  |  | *ref.* |  |  |  |
|  | Middle | 1.45 | -0.95 | 3.85 | 0.237 | 0.74 | -1.00 | 2.47 | 0.405 | 0.32 | -0.99 | 1.63 | 0.631 |
|  | Richest | 2.84 | 0.28 | 5.41 | 0.030 | 0.11 | -2.97 | 3.20 | 0.942 | 1.21 | -0.36 | 2.77 | 0.131 |
| Reports at least one danger sign | | 1.55 | 0.11 | 2.99 | 0.035 | -1.15 | -2.73 | 0.42 | 0.152 | 0.49 | -1.33 | 2.30 | 0.598 |
| Rates own health as poor or fair | | -1.66 | -2.98 | -0.34 | 0.013 | -0.48 | -3.24 | 2.28 | 0.734 | 2.36 | 0.71 | 4.02 | 0.005 |
| Primiparous | | 0.74 | -0.71 | 2.19 | 0.315 | -2.90 | -4.56 | -1.25 | 0.001 | -1.17 | -2.63 | 0.30 | 0.119 |
| Pregnancy was intended | | 0.46 | -1.50 | 2.41 | 0.646 | 1.49 | 0.64 | 2.33 | 0.001 | 0.74 | -1.20 | 2.68 | 0.456 |
| **Facility characteristics** | |  |  |  |  |  |  |  |  |  |  |  |  |
| Private |  | 0.91 | -3.21 | 5.03 | 0.666 | 2.86 | -6.17 | 11.89 | 0.535 |  |  |  |  |
| Secondary | | -4.84 | -9.76 | 0.08 | 0.054 | 4.96 | -2.19 | 12.11 | 0.174 | 2.46 | -2.78 | 7.70 | 0.358 |
| Service readiness | |  |  |  |  |  |  |  |  |  |  |  |  |
|  | Low | *ref.* |  |  |  | *ref.* |  |  |  | *ref.* |  |  |  |
|  | Middle | -1.38 | -7.41 | 4.65 | 0.653 | 7.87 | -5.98 | 21.72 | 0.265 | -2.05 | -7.24 | 3.13 | 0.438 |
|  | High | -1.18 | -7.01 | 4.65 | 0.692 | 3.56 | -13.96 | 21.09 | 0.690 | -4.63 | -9.75 | 0.48 | 0.076 |
| Number of staff providing obstetric care | |  |  |  |  |  |  |  |  |  |  |  |  |
|  | Low | *ref.* |  |  |  | *ref.* |  |  |  | *ref.* |  |  |  |
|  | Middle | -5.57 | -12.78 | 1.63 | 0.130 | -1.32 | -10.76 | 8.11 | 0.783 | -4.71 | -9.03 | -0.40 | 0.032 |
|  | High | 11.89 | 4.54 | 19.24 | 0.002 | -5.65 | -13.93 | 2.62 | 0.180 | -3.54 | -7.60 | 0.52 | 0.088 |
| Number of antenatal care visits per month | |  |  |  |  |  |  |  |  |  |  |  |  |
|  | Low |  |  |  |  |  |  |  |  | *ref.* |  |  |  |
|  | Middle |  |  |  |  |  |  |  |  | -0.76 | -4.84 | 3.32 | 0.715 |
|  | High |  |  |  |  |  |  |  |  | 0.00 | -4.34 | 4.33 | 0.999 |
| **Site** |  |  |  |  |  |  |  |  |  |  |  |  |  |
| Rural site | | *ref.* |  |  |  | *ref.* |  |  |  | *ref.* |  |  |  |
| Urban site | | -2.53 | -9.48 | 4.41 | 0.475 | 0.02 | -7.92 | 7.97 | 0.995 | -1.50 | -4.98 | 1.97 | 0.397 |
| **N** |  | **980** |  |  |  | **984** |  |  |  | **1012** |  |  |  |
| Intercept | | 39.40 | 34.14 | 44.65 | <0.001 | 60.20 | 53.71 | 66.69 | <0.001 | 78.48 | 73.08 | 83.88 | <0.001 |
|  |  | **South Africa** | | | |  |  |  |  |  |  |  |  |
|  |  | **Coeff** | **LCL** | **UCL** | **p-value** |  |  |  |  |  |  |  |  |
| **Risk factors** | |  |  |  |  |  |  |  |  |  |  |  |  |
| No risk factor | | *ref.* |  |  |  |  |  |  |  |  |  |  |  |
| One risk factor | | 0.23 | -1.96 | 2.42 | 0.836 |  |  |  |  |  |  |  |  |
| Two risk factors | | 0.55 | -1.97 | 3.07 | 0.669 |  |  |  |  |  |  |  |  |
| Three or more risk factors | | -0.18 | -2.97 | 2.60 | 0.898 |  |  |  |  |  |  |  |  |
| **Demographics** | |  |  |  |  |  |  |  |  |  |  |  |  |
| Age |  |  |  |  |  |  |  |  |  |  |  |  |  |
|  | 20-34 | *ref.* |  |  |  |  |  |  |  |  |  |  |  |
|  | < 20 | 0.57 | -1.87 | 3.01 | 0.646 |  |  |  |  |  |  |  |  |
|  | 35+ | 1.00 | -1.25 | 3.26 | 0.384 |  |  |  |  |  |  |  |  |
| Completed secondary school | | 0.87 | -0.57 | 2.30 | 0.237 |  |  |  |  |  |  |  |  |
| Answers health literacy questions correctly | | 1.31 | -0.02 | 2.63 | 0.054 |  |  |  |  |  |  |  |  |
| Wealth |  |  |  |  |  |  |  |  |  |  |  |  |  |
|  | Poorest | *ref.* |  |  |  |  |  |  |  |  |  |  |  |
|  | Middle | 1.62 | -0.18 | 3.43 | 0.077 |  |  |  |  |  |  |  |  |
|  | Richest | 1.00 | -0.72 | 2.73 | 0.254 |  |  |  |  |  |  |  |  |
| Reports at least one danger sign | | 0.99 | -0.36 | 2.35 | 0.151 |  |  |  |  |  |  |  |  |
| Rates own health as poor or fair | | 2.44 | 0.06 | 4.82 | 0.044 |  |  |  |  |  |  |  |  |
| Primiparous | | -1.06 | -2.75 | 0.64 | 0.221 |  |  |  |  |  |  |  |  |
| Pregnancy was intended | | 0.55 | -1.38 | 2.49 | 0.575 |  |  |  |  |  |  |  |  |
| **Facility characteristics** | |  |  |  |  |  |  |  |  |  |  |  |  |
| Private |  |  |  |  |  |  |  |  |  |  |  |  |  |
| Secondary | |  |  |  |  |  |  |  |  |  |  |  |  |
| Service readiness | |  |  |  |  |  |  |  |  |  |  |  |  |
|  | Low | *ref.* |  |  |  |  |  |  |  |  |  |  |  |
|  | Middle | 4.54 | 0.87 | 8.22 | 0.015 |  |  |  |  |  |  |  |  |
|  | High | 0.88 | -3.95 | 5.70 | 0.722 |  |  |  |  |  |  |  |  |
| Number of staff providing obstetric care | |  |  |  |  |  |  |  |  |  |  |  |  |
|  | Low | *ref.* |  |  |  |  |  |  |  |  |  |  |  |
|  | Middle | -3.32 | -7.57 | 0.92 | 0.125 |  |  |  |  |  |  |  |  |
|  | High | 1.80 | -7.83 | 11.43 | 0.714 |  |  |  |  |  |  |  |  |
| Number of antenatal care visits per month | |  |  |  |  |  |  |  |  |  |  |  |  |
|  | Low | *ref.* |  |  |  |  |  |  |  |  |  |  |  |
|  | Middle | -5.29 | -9.40 | -1.18 | 0.012 |  |  |  |  |  |  |  |  |
|  | High | -5.33 | -14.70 | 4.04 | 0.265 |  |  |  |  |  |  |  |  |
| **Site** |  |  |  |  |  |  |  |  |  |  |  |  |  |
| Rural site | | *ref.* |  |  |  |  |  |  |  |  |  |  |  |
| Urban site | | 2.04 | -2.15 | 6.23 | 0.340 |  |  |  |  |  |  |  |  |
| **N** |  | **1035** |  |  |  |  |  |  |  |  |  |  |  |
| Intercept | | 74.99 | 68.75 | 81.23 | <0.001 |  |  |  |  |  |  |  |  |

## **Supplemental Table E. STROBE Statement**

- checklist of items that should be included in reports of observational studies

|  | Item No | Recommendation | Section and paragraph number |
| --- | --- | --- | --- |
| **Title and abstract** | 1 | (*a*) Indicate the study’s design with a commonly used term in the title or the abstract | **First page** |
|  |  | (*b*) Provide in the abstract an informative and balanced summary of what was done and what was found | **First page** |
| Introduction | | | |
| Background/rationale | 2 | Explain the scientific background and rationale for the investigation being reported | **Introduction section** |
| Objectives | 3 | State specific objectives, including any prespecified hypotheses | **Introduction section** |
| Methods | | | |
| Study design | 4 | Present key elements of study design early in the paper | **Methods – data sources** |
| Setting | 5 | Describe the setting, locations, and relevant dates, including periods of recruitment, exposure, follow-up, and data collection | **Methods** |
| Participants | 6 | (*a*) *Cohort study*—Give the eligibility criteria, and the sources and methods of selection of participants. Describe methods of follow-up  *Case-control study*—Give the eligibility criteria, and the sources and methods of case ascertainment and control selection. Give the rationale for the choice of cases and controls  *Cross-sectional study*—Give the eligibility criteria, and the sources and methods of selection of participants | **Methods** |
|  |  | (*b*) *Cohort study*—For matched studies, give matching criteria and number of exposed and unexposed  *Case-control study*—For matched studies, give matching criteria and the number of controls per case |  |
| Variables | 7 | Clearly define all outcomes, exposures, predictors, potential confounders, and effect modifiers. Give diagnostic criteria, if applicable | **Methods** |
| Data sources/ measurement | 8* | For each variable of interest, give sources of data and details of methods of assessment (measurement). Describe comparability of assessment methods if there is more than one group | ***Methods*** |
| Bias | 9 | Describe any efforts to address potential sources of bias | **Methods and discussion (limitation) sections** |
| Study size | 10 | Explain how the study size was arrived at | **Methods** |
| Quantitative variables | 11 | Explain how quantitative variables were handled in the analyses. If applicable, describe which groupings were chosen and why | **Methods** |
| Statistical methods | 12 | (*a*) Describe all statistical methods, including those used to control for confounding | **Methods – statistical analysis** |
|  |  | (*b*) Describe any methods used to examine subgroups and interactions | **Methods – statistical analysis** |
|  |  | (*c*) Explain how missing data were addressed | **Methods – statistical analysis** |
|  |  | (*d*) *Cohort study*—If applicable, explain how loss to follow-up was addressed  *Case-control study*—If applicable, explain how matching of cases and controls was addressed  *Cross-sectional study*—If applicable, describe analytical methods taking account of sampling strategy | **N/A** |
|  |  | (*e*) Describe any sensitivity analyses | **Methods – statistical analysis** |

| Results | | | |
| --- | --- | --- | --- |
| Participants | 13* | (a) Report numbers of individuals at each stage of study—eg numbers potentially eligible, examined for eligibility, confirmed eligible, included in the study, completing follow-up, and analysed | **Methods and results sections** |
|  |  | (b) Give reasons for non-participation at each stage | **N/A** |
|  |  | (c) Consider use of a flow diagram | **N/A** |
| Descriptive data | 14* | (a) Give characteristics of study participants (eg demographic, clinical, social) and information on exposures and potential confounders | **Results and Table 1** |
|  |  | (b) Indicate number of participants with missing data for each variable of interest | **Table 2** |
|  |  | (c) *Cohort study*—Summarise follow-up time (eg, average and total amount) | N/A |
| Outcome data | 15* | *Cohort study*—Report numbers of outcome events or summary measures over time |  |
|  |  | *Case-control study—*Report numbers in each exposure category, or summary measures of exposure |  |
|  |  | *Cross-sectional study—*Report numbers of outcome events or summary measures | **Table 1, Figure 1-2** |
| Main results | 16 | (*a*) Give unadjusted estimates and, if applicable, confounder-adjusted estimates and their precision (eg, 95% confidence interval). Make clear which confounders were adjusted for and why they were included | **Results and table 2** |
|  |  | (*b*) Report category boundaries when continuous variables were categorized | **Table 1 and 2** |
|  |  | (*c*) If relevant, consider translating estimates of relative risk into absolute risk for a meaningful time period |  |
| Other analyses | 17 | Report other analyses done—eg analyses of subgroups and interactions, and sensitivity analyses | **Methods and Results** |
| Discussion | | | |
| Key results | 18 | Summarise key results with reference to study objectives | **Discussion section** |
| Limitations | 19 | Discuss limitations of the study, taking into account sources of potential bias or imprecision. Discuss both direction and magnitude of any potential bias | **Discussion section** |
| Interpretation | 20 | Give a cautious overall interpretation of results considering objectives, limitations, multiplicity of analyses, results from similar studies, and other relevant evidence | **Discussion section** |
| Generalisability | 21 | Discuss the generalisability (external validity) of the study results | **Discussion section** |
| Other information | | | |
| Funding | 22 | Give the source of funding and the role of the funders for the present study and, if applicable, for the original study on which the present article is based | **Submitted on portal** |

*Give information separately for cases and controls in case-control studies and, if applicable, for exposed and unexposed groups in cohort and cross-sectional studies.
